# Early antibiotic therapy is associated with a lower probability of successful liberation from mechanical ventilation in patients with severe acute exacerbation of chronic obstructive pulmonary disease

**DOI:** 10.1186/s13613-022-01060-2

**Published:** 2022-09-24

**Authors:** G. Deniel, M. Cour, L. Argaud, J. C. Richard, L. Bitker

**Affiliations:** 1 Service de Médecine Intensive - Réanimation, Hôpital de la Croix Rousse, Hospices Civils de Lyon, 103 Grande Rue de la Croix Rousse, 69004 Lyon, France; 2 Université de Lyon, Claude Bernard Lyon 1, Lyon, France; 3 INSA-Lyon, CNRS, INSERM, CREATIS UMR 5220, U1294 Villeurbanne, France; 4 Service de Médecine Intensive - Réanimation, Hôpital Edouard Herriot, Hospices Civils de Lyon, Lyon, France

**Keywords:** Acute exacerbation, Chronic obstructive pulmonary disease, COPD, Mechanical ventilation, Ventilation weaning

## Abstract

**Background:**

While antibiotic therapy is advocated to improve outcomes in acute exacerbation of chronic obstructive pulmonary disease (AECOPD) whenever mechanical ventilation is required, the evidence relies on small studies carried out before the era of widespread antibiotic resistance. Furthermore, the impact of systematic antibiotic therapy on successful weaning from mechanical ventilation was never investigated accounting for the competitive risk of death. The aim of the study was to assess whether early antibiotic therapy (eABT) increases successful mechanical ventilation weaning probability as compared to no eABT, in patients with AECOPD without pneumoniae, using multivariate competitive risk regression.

**Methods:**

Retrospective analysis of patients admitted in 2 intensive care units (ICU) from 2012 to 2020 for AECOPD without pneumonia and requiring mechanical ventilation. eABT was defined as any anti-bacterial chemotherapy introduced during the first 24 h after ICU admission. The primary outcomes were the adjusted subdistribution hazard ratio (SHR) of the probability of being successfully weaned from mechanical ventilation (i.e. non-invasive and invasive ventilation) according to eABT status and accounting for the competitive risk of death.

**Results:**

Three hundred and ninety-one patients were included, of whom 66% received eABT. eABT was associated with a lower probability of successful liberation from mechanical ventilation when accounting for the competing risk of death in multivariate analyses (SHR 0.71 [95% confidence interval, 0.57–0.89], *p* < 0.01), after adjustment with covariates of disease severity. This association was present in all subgroups except in patients under invasive mechanical ventilation on ICU day-1, in patients with ICU day-1 worst PaCO_2_ > 74 torr (median value) and in patients with a documented bacterial bronchitis at ICU admission. Ventilator-free days at day 28, ICU-free days at day 28 and invasive mechanical ventilation-free days at day 28, were significantly lower in the eABT group, while there was no significant difference in mortality at day 28 between patients who received eABT and those who did not.

**Conclusions:**

eABT was independently associated with a lower probability of being successfully weaned from mechanical ventilation, suggesting that the clinician decision to overrule systematic administration of eABT was not associated with a detectable harm in AECOPD ICU patients without pneumonia.

**Supplementary Information:**

The online version contains supplementary material available at 10.1186/s13613-022-01060-2.

## Introduction

Chronic obstructive pulmonary disease (COPD) is a major cause of death and morbidity worldwide [1, 2], with a substantial economic burden, accounting for almost 6% of the total annual healthcare budget in the European Union [3]. Acute exacerbation of COPD (AECOPD) is a severe complication of COPD, which may lead to alveolar hypoventilation, respiratory failure, and death. Severe AECOPD is a common cause of intensive care unit (ICU) admission [4], and is associated with substantial mortality (up to 8% at hospital discharge and above 20% at 1 year) [5, 6].

Infectious respiratory diseases are one of the main causes of AECOPD [7], but more than half of infectious-related AECOPD are caused by a viral pathogen [8]. Hence, up to 40% of AECOPD aetiologies are non-infectious [9], and many AECOPD patients are not expected to benefit from antibiotic therapy. On the other hand, delaying antibiotic initiation may be harmful in severe patients with documented bacterial infection. Yet, a single small randomized controlled trial published in 2001 (i.e. before widespread antimicrobial resistance) on mechanically ventilated ICU patients with AECOPD demonstrated a mortality benefit of systematic antibiotic therapy with ofloxacin, but these results were never confirmed [10]. In an era of antibiotic sparing, the relevance of systematic antibiotic therapy may be questioned [11, 12], especially since it may promote bacterial colonization and multi-drug resistant pathogens selection [13]. The use of biomarker-based strategies has been proposed [14–16], but a recent randomized controlled trial failed to demonstrate non-inferiority of a procalcitonin (PCT)-based algorithm to guide initial antibiotic therapy in ICU patients with AECOPD [17]. Hence, national and international guidelines still recommend systematic antibiotic use as soon as mechanical ventilation is required in ICU patients with AECOPD [1, 18].

We hypothesized that early antibiotic therapy at ICU admission of severe AECOPD patient would be associated with a higher probability of being successfully weaned from the ventilator. This endpoint may be more relevant than ICU mortality as mortality is low in AECOPD, and as early antibiotic administration was previously related to a decrease in mechanical ventilation duration in mechanically ventilated ICU patients with AECOPD [10].

The primary aim of the study was to evaluate whether early antibiotic therapy was associated with an increased probability of being liberated from mechanical ventilation, using multivariate competitive risk regression. The secondary objective of the study was to identify subgroups of patients in whom antibiotic therapy could be delayed or withheld without harm.

## Materials and methods

We conducted an observational, retrospective, non-matched case–control study in 2 French medical ICUs located in a university hospital. The study protocol was reviewed and approved by the Hospices Civils de Lyon ethics committee for human research (21_303), and complied with the STROBE criteria for observational studies [19]. Patients were informed by a postal letter of their enrolment in the study, and that they could oppose the use of their data.

### Inclusion criteria

Eligible participants were adults (> 18 years) presenting with AECOPD according to GOLD guidelines [1], with a thoracic imaging (chest radiography or thoracic CT scanner) performed within the first 24 h after ICU admission and with acute respiratory failure defined as:

- Either clinical respiratory distress (respiratory rate > 25/min and use of accessory muscles) associated with hypercapnic acidosis (pH < 7.35 and arterial carbon dioxide partial pressure (PaCO_2_) > 45 mmHg) and the use of non-invasive ventilation (NIV) for more than 4 h during the first 24 h after ICU admission;
- Or clinical respiratory distress requiring invasive mechanical ventilation on ICU day-1 (i.e. first calendar day of ICU admission).

### Exclusion criteria

Most of the criteria aimed at excluding patients for whom a cause of the AECOPD were identified. They were excluded if a pneumonia was diagnosed within the first 24 h of ICU admission (defined as the occurrence of a novel alveolar infiltrates and at least one of the following criteria: fever > 38.3 °C or hypothermia < 36 °C or polynuclear neutrophils > 7G/L or microbial documentation on respiratory sample). Other exclusion criteria were the following: non-COPD chronic respiratory disease (e.g., overlap syndrome, asthma, interstitial lung diseases), immunodeficiency, neuromuscular disease, trauma, admission from a non-participating ICU in which the patient stayed 48 h or more, non-pulmonary bacterial infection, pneumothorax or ICU admission for intoxication.

### Exposure to early antibiotic therapy

We defined early antibiotic therapy (eABT) as any anti-bacterial chemotherapy introduced during the first 24 h after ICU admission, regardless of previous treatment. Adequate eABT was defined as an antibiotic therapy effective on the documented bacteria, based on the antibiogram.

### Study outcomes

The primary outcome was the subdistribution hazard ratio (SHR) of eABT evaluating the probability of being liberated from mechanical ventilation during ICU hospitalization (i.e. non-invasive and invasive ventilation), after adjustment for relevant covariates and accounting for the competitive risk of death. Ventilation liberation was defined after 48 consecutive hours free of NIV. Date of ventilation liberation was the calendar day of the last NIV session before ventilation liberation. In chronically ventilated patients, it was defined as the return to usual NIV setting that were or will be applied outside ICU: same inspiratory and expiratory pressure levels, same NIV session number during the day, and same duration of each NIV session (with a maximum difference of plus or minus 2 h).

Secondary outcomes were ventilator-free days at day-28 (VFD), ICU-free days at day-28, hospital-free days at day-28, invasive mechanical ventilation-free days at day-28, mortality at day-28, and delayed invasive mechanical ventilation, defined as invasive mechanical ventilation initiated after ICU day-1. VFD were defined as the number of days without invasive or non-invasive ventilation (or return to pre-admission ventilatory settings in patients under home NIV). All free-days outcomes were set to 0 if the patient died between admission and day-28. The same was true if a patient was still receiving mechanical ventilation or was still in ICU or in hospital at day-28 after ICU admission.

### Respiratory sample, bacterial bronchitis and other respiratory events

We defined the variable “respiratory sample (presence/absence) at AECOPD onset” if a bacteriologic respiratory sample was performed between 48 h before and at the end of ICU day-1 (Additional file 1: Fig. S1). We considered this variable (if set to present) was an indication that the clinicians suspected bacterial infection, and had a possible impact on the eABT decision-making as the microbiological results could (at least partially) be available on ICU day-1. Documented bacterial bronchitis at admission was defined as the co-existence of a bacterial documentation (without pneumonia criteria as defined per the exclusion criteria) on a respiratory sample carried out at AECOPD onset as defined above. The thresholds for positive culture were: 10^5^ colony-forming units (CFU)/mL for sputum samples, 10^4^ CFU/mL for broncho-alveolar lavage (BAL) and 10^3^ CFU/mL for mini-BAL. *Corynebacterium* spp, *Neisseria *spp, *Enterococcus* spp, *Streptococcus viridans* and coagulase-negative *Staphylococcus* spp were not considered as pathogenic microorganisms.

Cardiogenic pulmonary oedema was identified if the electronic medical records reported statement of cardiogenic pulmonary oedema as the cause for AECOPD, or using the results of the trans-thoracic echocardiography (TTE) performed at ICU admission. The following TTE criteria were considered as evidence for high left ventricle filling pressures: 1- E-wave/A-wave > 2; 2- E-wave/A-wave between 0.8 and 2, or E-wave/A-wave < 0.8 and E > 0.5 m/s associated with at least two of three of the following criteria: lateral E-wave/e’-wave > 14, peak tricuspid regurgitation velocity > 2.8 m/s, left atrial maximal volume index > 34 mL.m^−2^ [20].

### Patient management

All patients underwent a thoracic imaging within the first 24 h of ICU admission. Antibiotic use and mechanical ventilation management (timing of initiation, ventilatory settings) were not protocolized in the two participating centres. PCT was mainly used by centre#1 to guide introduction and/or early cessation of antibiotic therapy, but was not protocolized during the study period. Weaning of invasive mechanical ventilation was protocol-guided in the 2 participating centres, based on the identification of clinical weaning criteria (reversal of acute respiratory failure, absence of or mild neurological or haemodynamic impairment), and the result of the spontaneous breathing test (performed daily with or without low-level of pressure support). NIV cessation was left at the clinician’s discretion in both centres.

### Data collection

The following variables were collected: age, sex, body mass index, Charlson score [21], GOLD score [1], forced expiratory volume over 1 s assessed in the last 6 months preceding ICU admission, multi-resistant bacterial or *Pseudomonas aeruginosa* respiratory colonization identified in the last 3 months preceding ICU admission, home oxygen or home NIV therapy). Patients were considered as frequent COPD exacerbators if they presented with more than 2 hospitalizations for AECOPD within a year. In addition, we collected the following variables on the day of ICU admission: arterial blood gas results, SOFA [22] and SAPS-2 [23] scores, PCT level, eABT class and duration of antibiotic therapy.

### Statistical analysis

All statistical analyses were performed using R software [24](version 4.0.5, with packages cmprsk [25], ggplot2 [26] and *mice* [27]). A *p* value < 0.05 was considered statistically significant. Missing values per variable are reported in Table 1. Missing data in multivariate analyses were handled using multiple imputations. Qualitative data are presented as counts (percentages) and their 95% confidence intervals (CI_95%_) according to the Wilson method if relevant, and quantitative data as median [interquartile range]. Comparisons between patients who received eABT or not were performed with the Fisher’s exact test for categorical variables, the t-test for continuous variables with normal distribution (assessed with Shapiro test), and the Wilcoxon–Mann–Whitney *U*-test for continuous variables that did not have a normal distribution. Multiple comparisons were performed using the Bonferroni–Holm method.

**Table 1:** Characteristics at ICU admission

Variables	Total population (*N* = 391)	Missing data	No eABT (*N* = 131)	eABT (*N* = 260)	*p*-value
Centre #1– no. (%)	186 (48%)	0 (0%)	71 (46%)	115 (56%)	0.07
Sex (female)—no. (%)	128 (33%)	0 (0%)	52 (40%)	76 (29%)	0.04
Age [IQR]—yr	71 [63–79]	0 (0%)	70 [61–80]	71 [64–78]	0.66
BMI [IQR]—kg.m^−2^	25 [19–29]	32 (8%)	25 [19–29]	24 [20–29]	0.69
Gold score—no. (%)		107 (27%)			0.55
1–2	46 (16%)		15 (16%)	31 (16%)	
3	92 (32%)		27 (28%)	65 (34%)	
4	146 (51%)		53 (56%)	93 (49%)	
Home oxygen—no. (%)	156 (40%)	0 (0%)	55 (42%)	101 (39%)	0.58
Home NIV—no. (%)	80 (20%)	0 (0%)	31 (24%)	48 (18%)	0.23
COPD frequent exacerbator—no. (%)	85 (22%)	0 (0%)	33 (25%)	52 (20%)	0.74
Active smoker—no. (%)	146 (43%)	53 (14%)	44 (39%)	102 (45%)	0.3
Charlson score [IQR]	5 [4–6]	0 (0%)	5 [3–6]	5 [4–6]	0.53
Invasive mechanical ventilation at ICU day-1—no. (%)	133 (34%)	0 (0%)	26 (20%)	107 (41%)	< 0.01
SAPS2 [IQR]	36 [30–46]	0 (0%)	33 (28–41)	37 (31–47)	0.001
*P. aeruginosa* colonization	33 (8%)	0 (0%)	10 (8%)	23 (9%)	0.85
ICU day-1 highest body temperature [IQR]—°C	37.2 [36.6–37.9]	0 (0%)	37 [36.7–37.5]	37.4 [37–38.2]	< 0.001
ICU day-1 worst Glasgow score without sedation	14 [11–15]	0 (0%)	14 [12–15]	14 [11–15]	0.14
ICU day-1 worst PaCO_2_ [IQR]—Torr	74 [63–89]	0 (0%)	76 [62.5–89]	74 [63–89]	0.98
ICU day-1 worst PaO_2_/FiO_2_ [IQR]—Torr		0 (0%)			0.01
>300	55 (14%)		28 (21%)	27 (10%)	
200–299	241 (62%)		77 (59%)	164 (63%)	
<200	95 (24%)		26 (20%)	69 (27%)	
ICU day-1 highest procalcitonin [IQR]—ng.mL^−1^	0.2 [0.1–0.5]	193 (49%)	0.1 [0.1–0.2]	0.3 [0.1–0.6]	< 0.001
Cardiogenic pulmonary oedema on ICU day-1—no. (%)	174 (45%)	0 (0%)	58 (44%)	116 (45%)	1
Antibiotic therapy before ICU day-1—no. (%)	105 (27%)	0 (0%)	21 (16%)	84 (32%)	< 0.01
IV steroid use within 24 h from ICU admission—no. (%)	88 (23%)	0 (0%)	33 (25%)	55 (21%)	0.37

eABT was defined as any anti-bacterial chemotherapy introduced during the first 24 h after ICU admission. COPD frequent exacerbator status was defined as at least two acute exacerbations of COPD with hospital admission within 1 year. Percentages were calculated according to population with complete data

*ICU* intensive care unit, *eABT* early antibiotic therapy, *BMI* body mass index, *NIV* non-invasive ventilation, *COPD* chronic obstructive pulmonary disease, *P. aeruginosa* *Pseudomonas aeruginosa*, *PaO*_*2*_*/FiO*_*2*_ O_2_ arterial partial pressure in arterial blood/fraction of inspired O_2_, *ICU* intensive care unit, SAPS2: Simplified Acute Physiology Score 2, *IV* intravenous and *IQR* interquartile range

Fine and Gray’s competing risk regression models were used to analyse the unadjusted subdistribution hazard ratios (SHRs) of each variable of interest associated with the probability of being successfully weaned from mechanical ventilation. Then, we created a multivariate model using the same methodology, including the following relevant variables: eABT status, centre, age, truncated SAPS2 (leaving out age, Glasgow Coma Scale, PaO_2_/FiO_2_ and temperature components to avoid collinearity with the other variables), home NIV or home oxygen status, COPD frequent exacerbator status, ICU day-1 cardiovascular SOFA subscore, ICU day-1 renal SOFA subscore, ICU day-1 highest body temperature, ICU day-1 worst Glasgow, ICU day-1 worst PaCO_2_, ICU day-1 worst PaO_2_/FiO_2_, invasive mechanical ventilation on ICU day-1, respiratory sample at AECOPD onset, cardiogenic pulmonary oedema on ICU day-1. Interactions and multicollinearity were systematically checked for, and the proportional hazard assumption was assessed by graphical analysis of the Schoenfeld residuals for each variable of interest. Then the model was finally reduced by deleting variables with *p*-value > 0.1.

Sensitivity analyses were conducted, using the reduced model as defined above and computing SHR for eABT in the following subgroups of patients: home NIV/home oxygen vs. no home NIV/home oxygen, ICU day-1 worst PaCO_2_ more or less than its median value (74 torr), positive respiratory sample for bacteria at AECOPD onset vs. negative respiratory sample for bacteria at AECOPD onset vs. no respiratory sample at AECOPD onset, invasive mechanical ventilation vs. no invasive mechanical ventilation on ICU day-1. In addition, we tested several definitions for eABT as antibiotic therapy introduced in the first 24, 48, or 72 h after ICU admission. Finally, we added PCT plasma concentrations to the reduced regression model, and performed an analysis restricted to patients of centre #1.

## Results

### Population

**Fig. 1:**
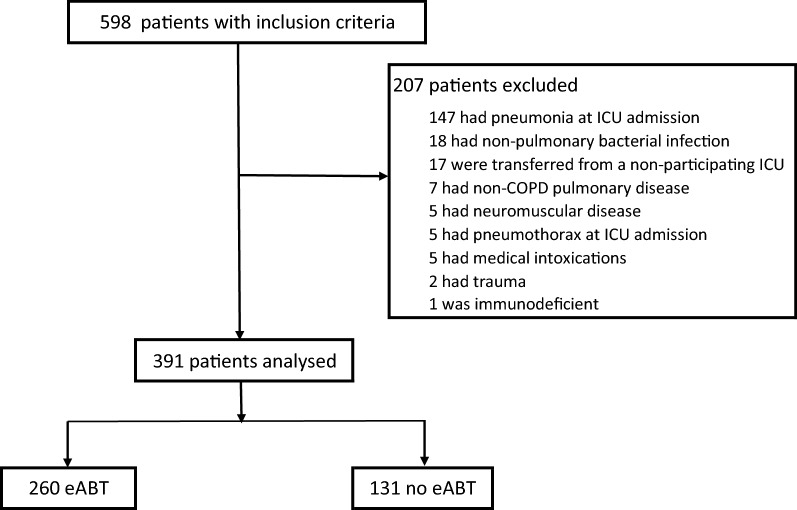
Patients screening. Screening lasted from July 2012 to January 2020. eABT was defined as the first line of antibiotic therapy introduced during the first 24 h after ICU admission. *eABT* early antibiotic therapy, *ICU* intensive care unit

From July 2012 to January 2020, 391 patients were enrolled in the study (Fig. 1). The main reason for exclusion was pneumonia (71% of excluded patients). Patients’ characteristics at ICU admission are provided in Table 1. Most of the patients were male (*n* = 263) with a median age of 71 years, and baseline disease severity was high with 146 patients (51%) presenting with a GOLD score of 4. A thoracic CT scanner was available within 24 h after ICU admission for 137 patients.

### Exposure

Among all included patients, 260 received an eABT. In the other group (No eABT), 39/131 (30%) received antibiotics after ICU day 1, within a median delay of 4 (3–5) days. A bacterial bronchitis was microbiologically documented at AECOPD onset in 63 patients (16%, CI_95%_, 12–20%), while 102 patients (26%, CI_95%_, 22–30%) did not have any respiratory sample at AECOPD onset. eABT classes and microbial documentations are summarized in Additional file 1: Fig. S2. Two hundred patients (51%, CI_95%_, 46–56%) received an eABT but did not have documented bacterial bronchitis. Only 42 (11%, CI_95%_, 8–14%) received an adequate eABT (Additional file 1: Fig. S3A). eABT duration was slightly but significantly shorter when no bacterial bronchitis was documented on respiratory sample at AECOPD onset (5 [3–7] days) as compared with patients without respiratory sample at AECOPD onset (6 [4–8] days), or with documented bronchitis (7 [5–10] days) (Additional file 1: Fig. S3B). Patients treated with eABT presented with significantly greater clinical severity, as compared to patients who did not, regarding SAPS2, ICU day-1 highest body temperature and worst PaO_2_/FiO_2_ ratio (Table 1).

### Primary outcome

**Fig. 2:**
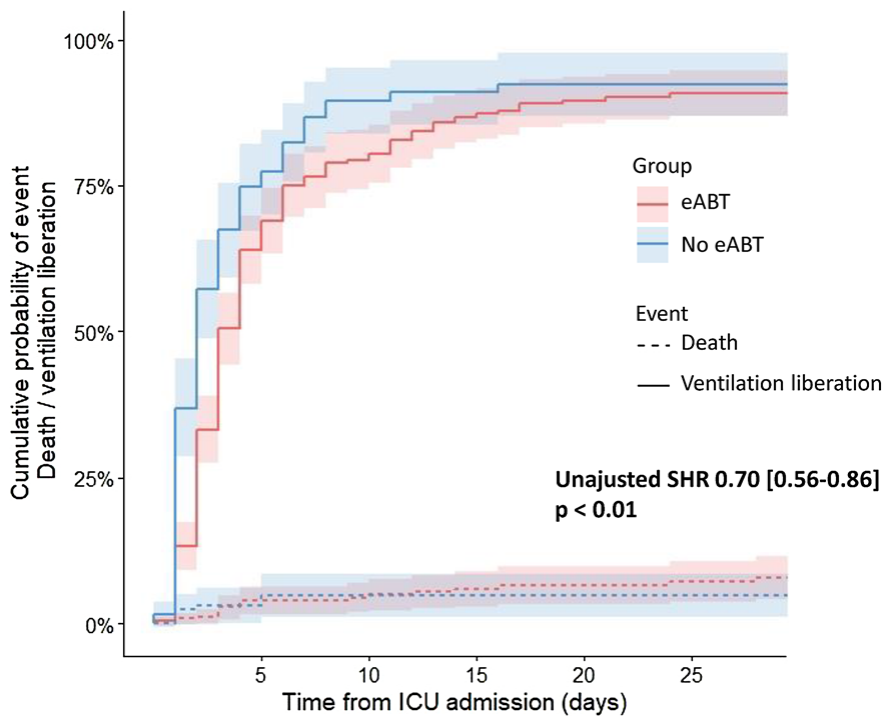
Cumulative incidence of successful weaning from mechanical ventilation and death according to eABT. eABT was the first line of antibiotic therapy introduced during the first 24 h of ICU admission. Continuous lines are cumulative incidence function curves of the probability of being successfully weaned from mechanical ventilation modelled with univariate Fine and Gray regression accounting for the competitive risk of death, with eABT as the independent variable. Broken lines are cumulative incidence function curves of the probability of dying in the ICU. Shaded areas are 95% confidence intervals. *eABT* early antibiotic therapy, *SHR* subdistribution hazard ratio, *CI95%* 95% confidence interval, *ICU* intensive care unit

**Fig. 3:**
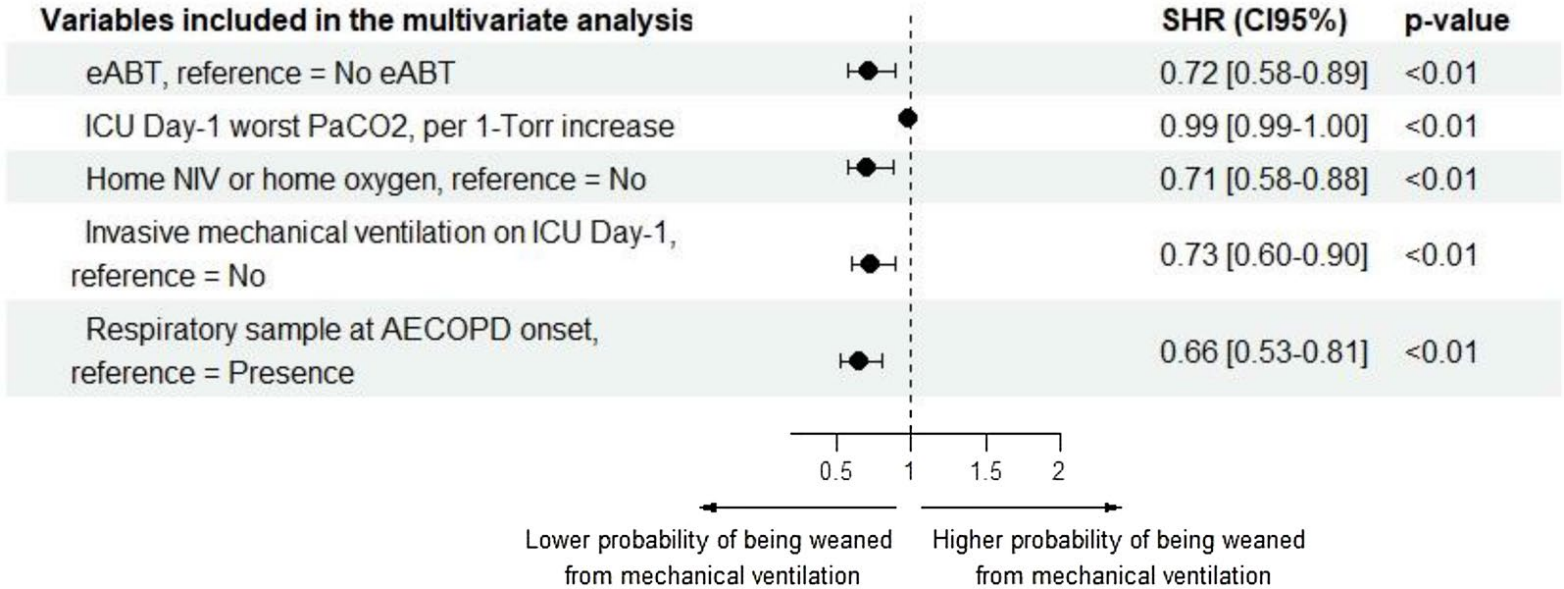
Forest plot of the adjusted SHR computed with multivariate computing risk regression of the probability of being successfully weaned from mechanical ventilation*.* eABT was defined as the first line of antibiotic therapy introduced during the first 24 h of ICU admission. COPD frequent exacerbator status was defined as at least two acute exacerbations of COPD with hospital admission within one year. Respiratory sample at AECOPD onset was defined as bacterial respiratory sample performed between 48 h before ICU admission and the end of ICU day-1. Univariate Fine and Gray analysis was first performed on each variable of interest (Additional file 1: Table S1). Then, the following variables were selected for inclusion in the multivariate model because of their assumed relevance: eABT status, centre, age, truncated SAPS2 (leaving out age, Glasgow Coma Scale, PaO2/FiO2 and temperature components to avoid collinearity with the other variables), home NIV or home oxygen status, COPD frequent exacerbator status, ICU day-1 cardiovascular and renal SOFA subscore (to avoid multicollinearity with SAPS2), ICU day-1 highest body temperature, ICU day-1 worst Glasgow, ICU day-1 worst PaCO2, ICU day-1 worst PaO2/FiO2 (PaO2/FiO2 was entered as a categorical variable because it was reported as a SAPS2 value when data were collected), invasive mechanical ventilation on ICU-day-1, respiratory sample at AECOPD onset, and cardiogenic pulmonary oedema on ICU day-1. Interactions were systematically checked for. We did not report any significant interaction between eABT and other variables included in our model. Then, we reduced our model by deleting all variables with multivariate p-value > 0.1. SHR lower than 1 indicates a lower probability of being successfully weaned from mechanical ventilation, accounting for the competing risk of death. *SHR* subdistribution hazard ratio; *CI95%* 95% confidence interval, *NIV* non-invasive ventilation, *PaCO2* CO2 arterial partial pressure; *PaO2/FiO2* ratio of O2 arterial partial pressure on fraction of inspired O2, *SAPS2* simplified acute physiology score 2, *SOFA* Sequential Organ Failure Assessment, *eABT* early antibiotic therapy, *COPD* chronic obstructive pulmonary disease, *ICU* intensive care unit

eABT was associated with a significantly lower probability of being successfully weaned from mechanical ventilation (accounting for the competitive risk of death) in both univariate and multivariate analyses (adjusted SHR amounting to 0.72 [CI_95%_, 0.58–0.89], Figs. 2 and 3, Additional file 1: Table S1). We did not report any interaction between eABT and other variables included in our model. The following variables were also independently related to a lower probability of being successfully weaned from mechanical ventilation: ICU day-1 worst PaCO_2_, home NIV or home oxygen, invasive mechanical ventilation on ICU day-1, and absence of respiratory sample at AECOPD onset.

### *Secondary outcomes (Table **2**)*

**Table 2:** Secondary outcomes

Variables	Total population (N = 391)	Missing data	No eABT (N = 131)	eABT (N = 260)	*p*-value
Ventilator-free days at day-28 [IQR]—day	25 [22–26]	0 (0%)	26 [22–27]	24 [21–26]	< 0.001
Day-28 mortality—no. (%)	31 (8%)	0 (0%)	9 (7%)	22 (8%)	0.69
ICU-free days at day-28 [IQR]—day	23 [20–25]	0 (0%)	24 [21–26]	23 [20–25]	0.001
Hospital-free days at day-28 [IQR]—day	13 [2–18]	76 (19%)	14 [6–20]	13 [4–17]	0.05
Invasive mechanical ventilation-free days at day-28 [IQR]—day	28 [26–28]	0 (0%)	28 [27, 28]	28 [25–28]	< 0.001
Delayed invasive mechanical ventilation—no. (%)	6 (2%)	0 (0%)	2 (2%)	4 (2%)	1

Ventilator-free days were defined as number of days without any respiratory assistance, invasive or non-invasive (or return of premorbid ventilation parameters in case of chronically ventilated patients). If the patient died at any time between admission and day-28, VFD were set to 0. ICU-free days and invasive ventilation-free days were number of days outside of the ICU or without invasive mechanical ventilation at day-28. Delayed invasive mechanical ventilation was defined as invasive mechanical ventilation onset more than 48 h after ICU admission

*ICU* intensive care unit, *eABT* early antibiotic therapy, and *IQR* interquartile range

At day-28 of ICU admission, 5 patients were still under mechanical ventilation. VFD, ICU-free days and invasive mechanical ventilation-free days were significantly lower in the eABT group. In the subgroup of patients with documented bacterial bronchitis at AECOPD onset (*N* = 63), VFD remained significantly lower in the eABT group, compared to unexposed patients (Additional file 1: Fig. S4).

### Sensitivity analyses

**Fig. 4:**
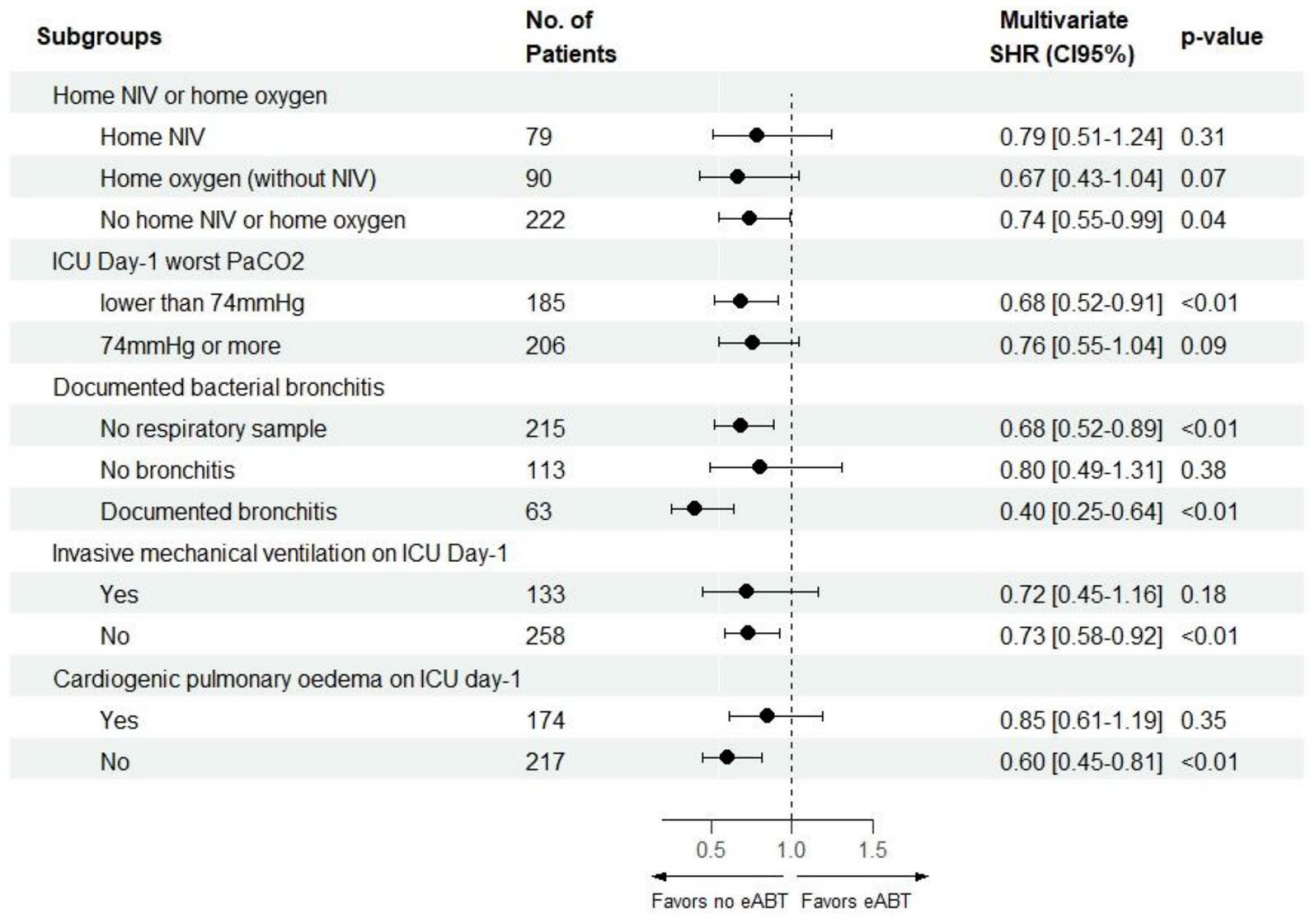
Sensitivity analysis of the SHR of eABT in subgroups of patients assessed by modelling the probability of being successfully weaned from mechanical ventilation using multivariate competing risk regression. eABT was the first line of antibiotic therapy introduced during the first 24 h of ICU admission. Documented bacterial bronchitis was defined as bacterial documentation on respiratory sample performed between 48 h before ICU admission and the end of ICU day-1. Seventy-four Torr was the median value of ICU Day-1 worst PaCO2. SHR less than 1 indicates a lower probability of being successfully weaned from mechanical ventilation, accounting for the competing risk of death. *SHR* subdistribution hazard ratio, *CI95%* 95% confidence interval, *eABT* early antibiotic therapy, *COPD* chronic obstructive pulmonary disease, *NIV* non-invasive ventilation, *ICU* intensive care unit

eABT was significantly associated with a decreased probability of being successfully weaned from mechanical ventilation in all subgroups, except in patients under invasive mechanical ventilation on ICU day-1, those with ICU day-1 worst PaCO_2_ > 74 torr and those without bacterial bronchitis based on respiratory sample performed at AECOPD onset (Fig. 4). Changes in the definition of the eABT time frame (from 24 to 72 h after ICU admission) did not affect the results of the primary analysis (Additional file 1: Fig. S5).

When PCT levels were added to the multivariate competing risk regression model built to evaluate the primary outcome and restricted to patients of centre #1, eABT was still significantly associated with a lower probability of being weaned from mechanical ventilation (SHR 0.65 [0.46–0.92], *p* = 0.02), while PCT levels were not (SHR 1.04 [0.99–1.09], *p* = 0.09).

## Discussion

We conducted a retrospective two-centre study evaluating the association of early antibiotic therapy on the probability of being successfully weaned from mechanical ventilation in ICU patients with severe AECOPD and without pneumonia. The main findings of the study were that: (1) 34% of the population patients did not receive eABT in opposition with international guidelines; (2) the probability of being successfully weaned from mechanical ventilation was significantly and independently lower in the eABT group; and (3) we were unable to identify a subgroup of patients in which eABT was associated with a more favourable outcome, as compared to a delayed strategy regarding antibiotic administration, including those with documented bacterial bronchitis at AECOPD onset.

We did not observe any benefit associated with eABT in terms of ventilation liberation probability. Likewise, we did not identify any evidence of harm in patients without eABT regarding the primary and secondary endpoints. These results are in marked contrast with previous studies [28, 29]. However, the number of prospective interventional studies evaluating the impact of systematic antibiotic therapy in COPD patients is scarce, with most studies conducted before 2001, and a single randomized controlled trial performed in the ICU setting [10]. Although the eligibility criteria of this randomized controlled trial by Nouira et al. was closed to ours, this study showed a significant decrease in mechanical ventilation duration (5.1 days to 3.1 days) and ICU mortality (17% to 4%) when once-daily ofloxacin was used as an early therapy in AECOPD ICU patients who required mechanical ventilation [10]. However, while patients in the Nouira trial were slightly less severe in terms of SAPS II score and rate of pre-inclusion home oxygen requirement, the rate of invasive mechanical ventilation during ICU hospitalization was substantially higher in their study (86% vs 36%), which may explain a mortality rate of nearly 20% in their placebo arm (more than twice the mortality rate of our study), in addition to improvement in general care of ICU patients during the last two decades. Furthermore, this study used an unusually low dose of ofloxacin for ICU patients (200 mg/day), and was performed before the era of widespread antimicrobial resistance and MDR pathogens. Indeed, nearly one in five bacterial bronchitis in our study were due to *Pseudomonas aeruginosa*, while it accounted for less than 5% of detected pathogens in Nouira’s study. We also reported more than 3% of inadequate eABT, which probably has a non-negligible impact in terms of emergence of antimicrobial resistance and may even be associated with poor outcomes. Furthermore, the same team conducted a single-centre retrospective study on 440 patients, and failed to identify a significant association of empiric antibiotic therapy initiated at ICU admission with ICU mortality [30]. The main reason given by the authors for the discrepancy in their studies results is the substantial increase in the use of NIV (from 29% in 2000 to 96.7% in 2012). Taken together, these studies and ours suggest that the beneficial impact of early antibiotherapy should be reassessed in the modern ICU setting, and that alternative endpoints to ICU mortality, such as time to successful weaning from mechanical ventilation should be considered. Interestingly, our study is the first to have addressed the impact of eABT on the probability of being weaned from mechanical ventilation, using appropriate statistical tools considering the competitive risk of death.

Surprisingly, eABT was significantly associated with a lower probability of ventilation liberation in the main analysis, and we did not identify any subgroups in which eABT was beneficial (including the group with documented bronchitis at AECOPD onset). We cannot exclude with certainty that this result is not the consequence of an unaccounted-for confounding variable. However, the competitive risk regression model was built with known parameters associated with severity of disease and outcome in patients with AECOPD. Also, all performed sensitivity analysis further confirmed the persistence of this association. We hypothesize that eABT could be associated with drug-related severe side effects, that may directly or indirectly prolong ventilation duration. Moreover, eABT may bear the risk of generating MDR pathogen colonization, and subsequently increase the risk of treatment failure of healthcare-acquired infections, including pneumonia. Supporting our findings, a recently published large meta-analysis evaluating the impact of PCT-guided antibiotic therapy in patients with acute respiratory tract infections reported a significative decrease in day-30 mortality in the PCT-guided strategy group [31]. This decrease in mortality was associated with significant decreases in antibiotic initiation, in their duration of administration, and in the incidence of antibiotics’ side effects, in the PCT-guided strategy group. Although we acknowledge that PCT-guided strategies intrinsically differ from eABT as defined in the present study, this work strongly suggests the potential innocuity of systematic antibiotic therapy introduction, and its possible consequences on patient outcome, including short-term survival. However, the meta-analysis did not report mechanical ventilation duration in the 2447 enrolled critically ill patients, limiting a more thorough comparison. On the other hand, we were unable to report neither eABT-related side effects, nor MDR pathogen colonization incidence after eABT exposure in our work, limiting our capacity to draw firm conclusions in this regard.

Despite AECOPD being a very common disease, randomized controlled trials on antibiotic therapy are scarce even in the non-ICU population. In the most recent meta-analysis in the AECOPD population, only 19 studies (enrolling 2663 patients) were included [12], but interpretation was hindered by the fact that most of them used different definitions for treatment failure. Only 2 trials reported mortality in hospitalized non-ICU patients and this endpoint was not statistically significant, while non-invasive ventilation duration was not reported [15, 16]. In probably what is the largest retrospective study in the field (84 621 patients), Rothberg et al. identified a significant decrease in mortality from 1.6% to 1.0% associated with early therapy after hospital admission as opposed to late (after 48 h) or no antibiotic therapy [28]. Of note, less than 1% of included patients were hospitalized in the ICU, in line with the observed low mortality rate. Whether these results observed in hospitalized non-ICU patients may apply to ICU patients is doubtful as ICU environment with close monitoring, easy access to reliable chest imaging techniques (CT, ultrasound) to exclude pneumonia and 24-h medical presence may allow to tailor individualized strategy with delayed antibiotic introduction according to clinical evolution or bacterial documentation based on respiratory sample.

eABT use was frequent in our study, but far from systematic as only 66% of the patients did not receive antibiotics, a rate similar to the one (59%) observed in a recent study on the same population [30]. Furthermore, the latter study reported an even greater decrease over time in the use of empiric antibiotic therapy in ICU patients admitted with AECOPD between 2000 and 2012 (from 68 to 33%) [30], along with a decreasing trend in mortality (from 12.9% in 2000 to 6.7% in 2012). This suggests that the ICU clinicians do not adhere to international guidelines regarding systematic antibiotic therapy in mechanically ventilated patients, at it may be associated adverse treatment effects as discuss above.

Regarding clinical implication, our study was observational, and focused on ICU patients in which pneumonia was ruled out by chest imaging. Patients with pneumonia or those with sepsis-related organ failure should be treated with antibiotics without delay, as this may impact survival [32]. Considering the available evidence and the fact that the absence of respiratory sample on ICU day-1 was associated with a lower probability of being weaned from mechanical ventilation in our work, clinicians should make every effort to document and treat bacterial bronchitis, using all available tools (direct analysis of respiratory sample, biomarkers) to ascertain respiratory tract bacterial infections. New tools such as multiplex polymerase chain reaction assay panel (mPCR) can provide bacterial or viral documentation with acceptable sensitivity and specificity in less than six hours [33], and could help eABT decision-making. However, the impact of PCT in the specific setting of ICU patients with AECOPD remains unclear when opposed to short-course antibiotic therapy with immediate cessation in case of negative bacterial samples [17]. In the absence of evident bacterial infection, early antibiotic treatment may prove potentially futile or even deleterious, in relation with treatment adverse events or MDR pathogen selection, which may in turn, be associated with prolonged ICU and hospital stays and worse clinical outcome [34]. However, the level of evidence of our work remains too low to modify international and national guidelines. Randomized control trials are required to validate our assumptions.

This study has the following strengths. It is the first study about antibiotics and AECOPD in ICU which focused on ventilation weaning, and reported the second largest cohort to date. Furthermore, it was a two-centre study and the large number of patients allowed inclusion of a large amount of variables in the multivariate model. Finally, we used an advanced statistical methodology, taking into account the competitive risk of death and that included variables known by the clinician at admission.

There are several limitations in our studies too. First, its retrospective design. Second, the use of the variable “respiratory sample at AECOPD onset” in our model is disputable as the information for the clinician is probably different whether the sample was performed 48 h before ICU admission or on ICU day-1 (Gram stain, positive culture). Third, we did not report treatment of the patients before ICU admission. Fourth, antibiotic-related adverse effects which may be part of explanation of lower ventilator liberation probability in the eABT group were not reported. Fifth, it remains possible that we did not identify a specific variable which explains association between eABT and lower probability of being successfully weaned from mechanical ventilation. Finally, there may be no correlation between eABT and ventilator liberation despite the association we described above.

## Conclusion

Early antibiotic therapy of patients admitted to the ICU with acute exacerbation of COPD without pneumonia and requiring mechanical ventilation was associated with a significantly lower probability of being successfully weaned from the ventilator, after adjustment for baseline COPD severity and severity of acute exacerbation. Our results suggest that the clinician decision to overrule systematic administration of eABT is not associated with a detectable harm in this setting, and that the future development of tools helping eABT decision-making may improve EACOPD prognosis and reduce antibiotic exposure.

## Supplementary Information

**Additional file 1**. Additional figures and tables.

## Data Availability

Data will be made available upon reasonable request.
