# Supplementary material for: Early antibiotic therapy is associated with a lower probability of successful liberation from mechanical ventilation in patients with severe acute exacerbation of chronic obstructive pulmonary disease
Source: Ann Intensive Care. 2022 Sep 24;12:86. doi: 10.1186/s13613-022-01060-2 (PMC9509513; doi:10.1186/s13613-022-01060-2)
Supplement: Supplementary file 1 — Additional file 1. Additional figures and tables. [file 13613_2022_1060_MOESM1_ESM.pdf]

ADDITIONAL DATA

Figure S1. Study time frame 2

Figure S2. Early antibiotics class and documented bacterial bronchitis at admission 3

Figure S3. Early antibiotic therapy and its duration as a function of microbial documentation in respiratory samples 4

Figure S4. VFD and invasive mechanical ventilation-free days as a function of eABT status in the 63 patients with documented bacterial bronchitis at AECOPD onset 5

Table S1. *Univariate competing risks analysis of the probability of being successfully weaned from respiratory support* 7

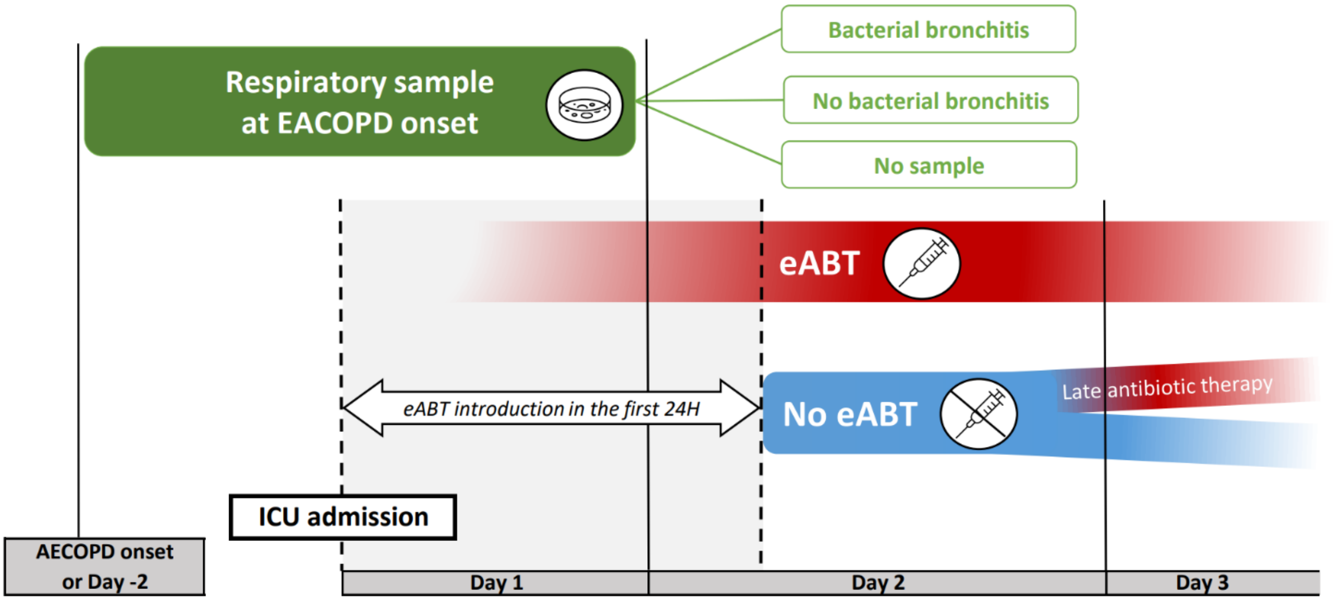

Figure S1. Study time frame

eABT was defined as the first line of antibiotic therapy introduced during the first 24h after ICU admission. We defined the variable "respiratory sample (presence/absence) at AECOPD onset" if a bacteriologic respiratory sample was performed between 48h before and at the end of ICU day-1 (i.e., first calendar day of ICU admission). AECOPD: acute exacerbation of chronic

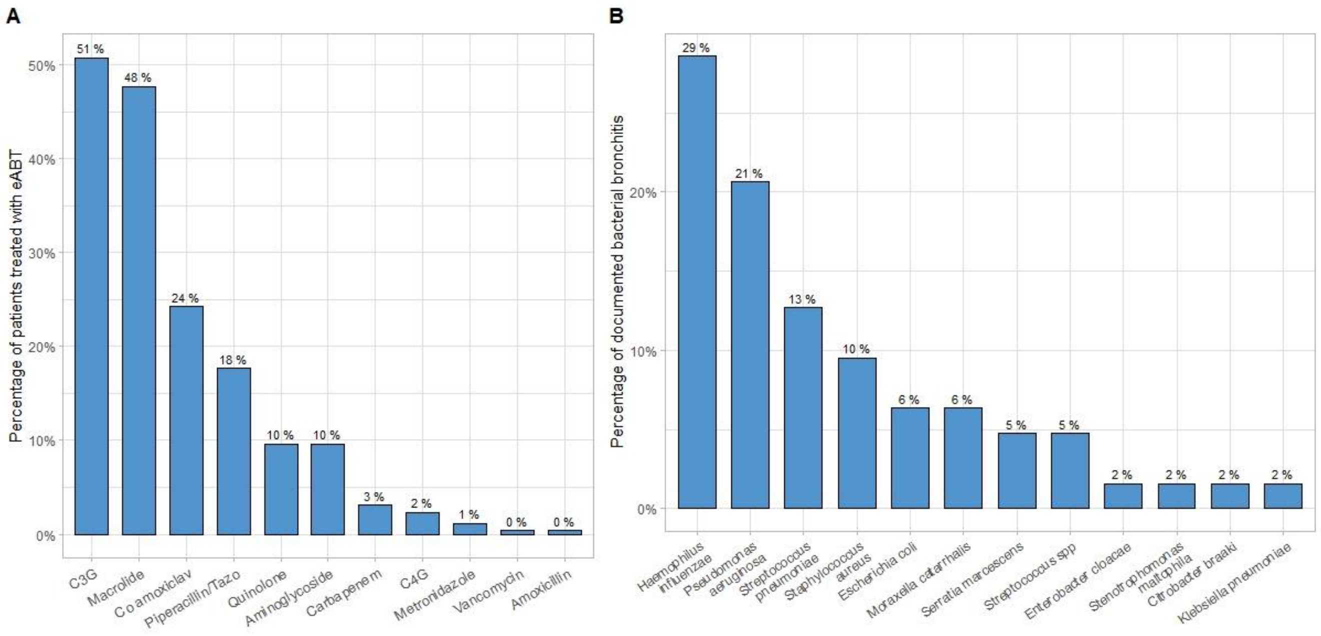

Figure S2. Early antibiotics class and documented bacterial bronchitis at admission

Panel A shows the percentage of antibiotic molecules in patients treated with eABT. eABT was defined as the first line of antibiotic therapy introduced during the first 24h of ICU admission. Percentages adds up above 100% as multiple antibiotics could be administered per patient. Panel B shows the percentage bacteria documented within the timeframe lasting from 48h before to ICU admission day. *eABT: early antibiotic therapy; C3G: third generation cephalosporin; C4G: fourth generation cephalosporin; ICU: intensive care unit; Tazo: tazobactam; spp: species.*

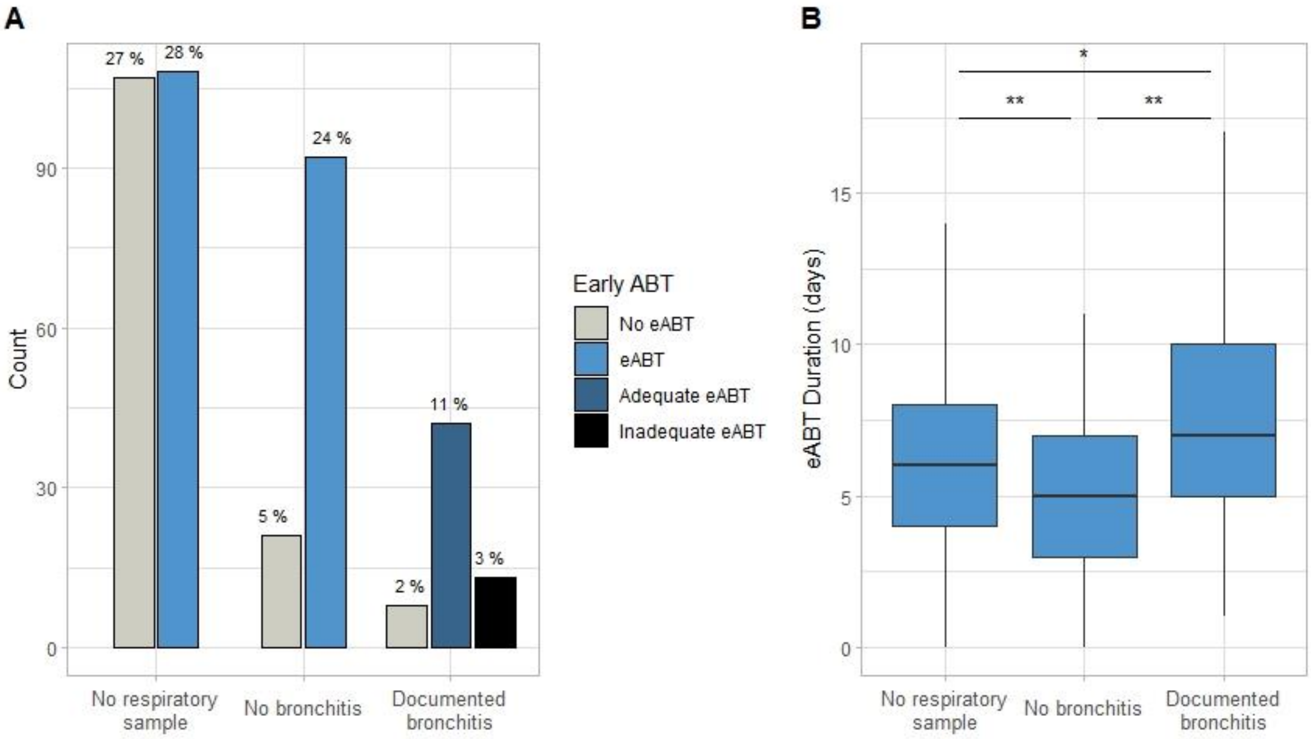

Figure S3. Early antibiotic therapy and its duration as a function of microbial documentation in respiratory samples

eABT was defined as any anti-bacterial chemotherapy introduced during the first 24h after ICU admission. Bacterial bronchitis documentation was considered within the timeframe lasting from 48h before ICU admission to ICU day-1. Panel A shows eABT as a function of microbial documentation. Adequate eABT was defined as an antibiotic therapy effective on the documented bacteria, based on the antibiogram. Panel B shows eABT duration as a function of microbial documentation. Percentages are calculated according to the total population count.

eABT: early antibiotic therapy.

** p-value <0.05, ** p-value <0.01 (Wilcoxon-Mann-Whitney U-test corrected with Bonferroni-Holm method for multiple testing)*

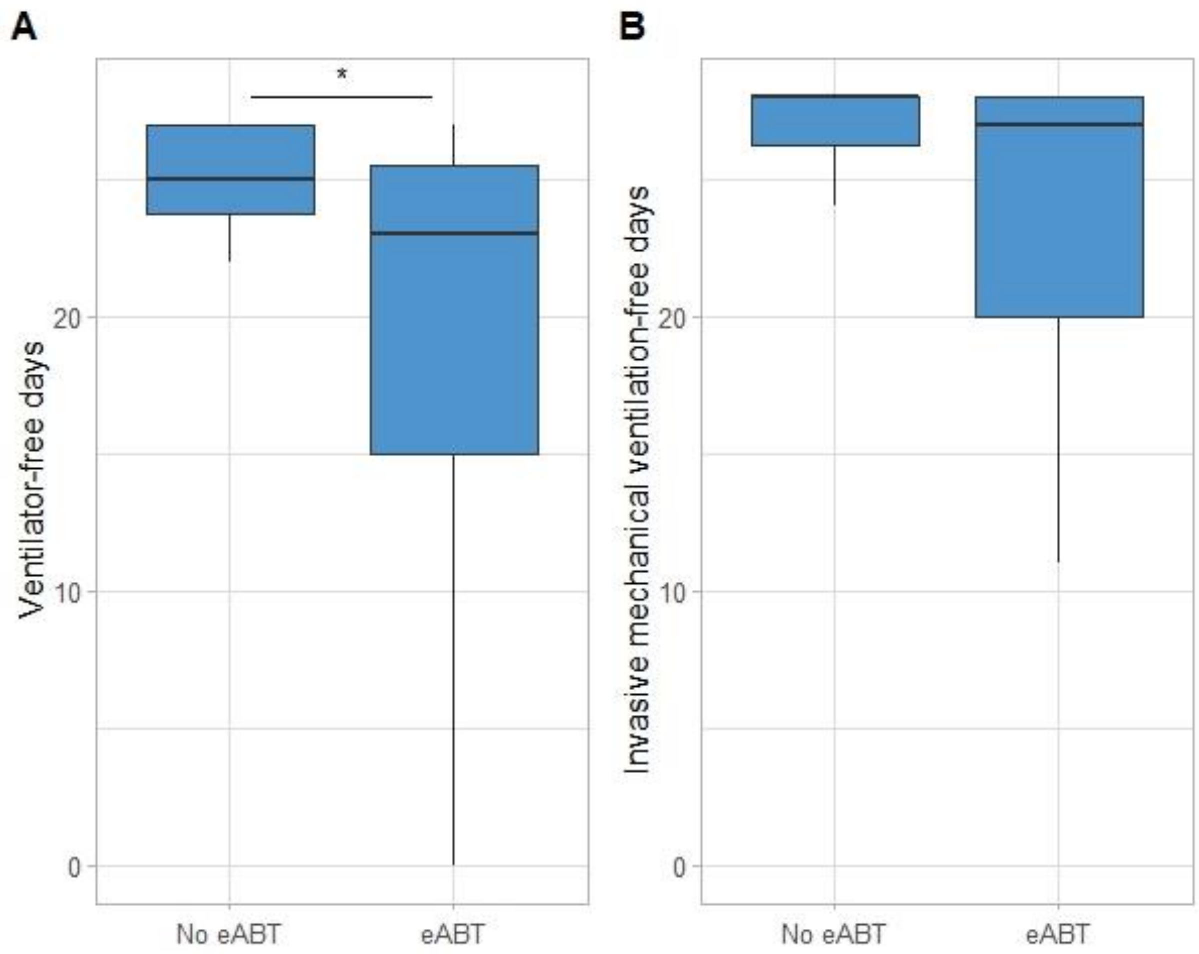

Figure S4. VFD and invasive mechanical ventilation-free days as a function of eABT status in the 63 patients with documented bacterial bronchitis at AECOPD onset

eABT was the first line of antibiotic therapy introduced during the first 24h of ICU admission. VFD are defined as the number of days without any respiratory assistance (invasive and non-invasive) during the first 28 days in ICU. VFD were set to zero if the patient died within this time frame. Same definition was applied for invasive ventilation-free days. Panel A shows VFD as a function of eABT use. Panel B shows invasive mechanical ventilation-free days as a function of eABT use.

VFD denotes ventilation free days, eABT early antibiotic therapy and ICU intensive care unit.

** p-value < 0.05*

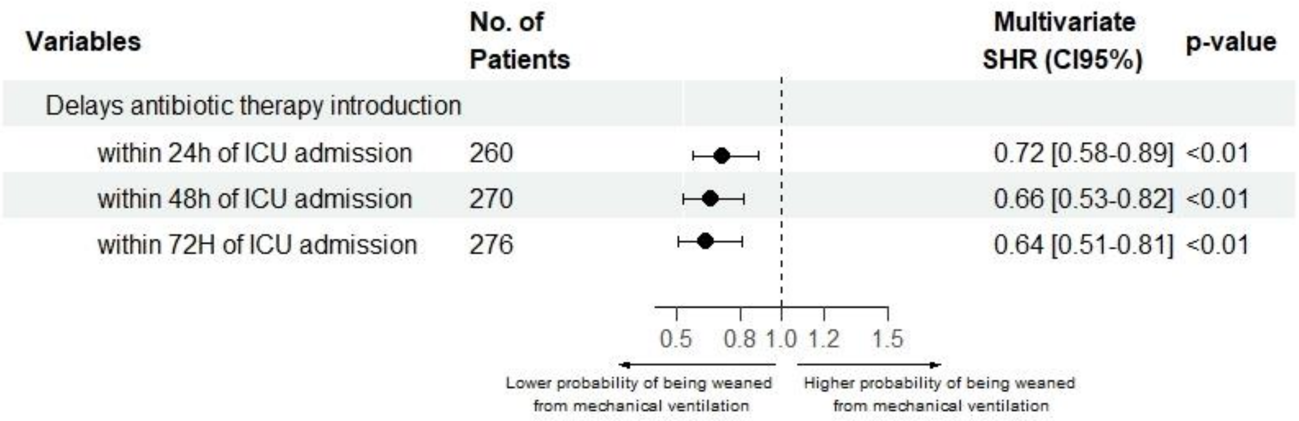

Figure S5. Sensitivity analysis of the SHR of eABT using modified eABT definition

eABT was the first line of antibiotic therapy introduced during the first 24h of ICU admission. eABT variable was modified in this analysis. We modified eABT definition as following: antibiotic therapy introduced 24h or less, 48h or less, 72h or less after ICU admission. eABT SHR from modified final Fine and Gray model are reported. SHR less than 1 indicates a lower probability of being successfully weaned from mechanical ventilation.

ICU denotes intensive care unit, SHR subdistribution hazard ratio, $CI_{95\%}$ *95% confidence interval and eABT early antibiotic therapy.*

Table S1. Univariate competing risks analysis of the probability of being successfully weaned from respiratory support

**Variables**	**Univariate SHR [$CI_{95\%}$]**	**Univariate p value**
Center #2, reference = center#1	0.89 [0.73-1.08]	0.24
Male gender, reference = female	0.89 [0.73-1.09]	0.28
Age, per 1-yr increase	1.00 [0.99-1.01]	0.89
Charlson score, per 1-point increase	1.02 [0.98-1.06]	0.42
Home NIV or home oxygen status, reference = No	0.71 [0.59-0.86]	<0.01
COPD frequent exacerbator status, reference = No	1.16 [0.93-1.45]	0.19
Truncated SAPS 2, per 1-point increase *	0.98 [0.97-0.99]	0.01
ICU Day-1 CV SOFA, per 1-point increase	0.94 [0.89-1.00]	0.07
ICU Day-1 renal SOFA, per 1-point increase	1.02 [0.92-1.13]	0.73
ICU Day-1 highest body temperature, per 1-°C increase	0.98 [0.89-1.08]	0.71
ICU Day-1 worst Glasgow, per 1-point increase	1.04 [1.01-1.07]	<0.01
ICU Day-1 worst $PaCO_2$, per 1-Torr increase	0.99 [0.98-0.99]	<0.01
ICU Day-1 worst PaO_2/FiO_2 >200 Torr, reference = $PaO_2/FiO_2 \leq 200$ Torr	0.72 [0.58-0.90]	<0.01
Antibiotics before ICU admission, reference = No	0.99 [0.79-1.22]	0.89
eABT, reference = No eABT	0.70 [0.56-0.86]	<0.01
Invasive mechanical ventilation on ICU Day-1, reference = No	0.68 [0.56-0.82]	<0.01
Bacterial bronchitis before ICU admission, reference = No	1.09 [0.52-2.30]	0.82
Respiratory sample at AECOPD onset, reference = presence	0.68 [0.56-0.84]	<0.01
Cardiogenic pulmonary oedema on ICU Day-1, reference = No	1.13 [0.94-1.36]	0.19

* Truncated SAPS2 was computed leaving out age, Glasgow coma scale, PaO_2/FiO_2 and temperature components to avoid collinearity with the other variables in the model building process.

eABT was defined as the first line of antibiotic therapy introduced during the first 24h of ICU admission.

COPD frequent exacerbator status was defined as at least two acute exacerbations of COPD with hospital admission within one year. Respiratory sample at AECOPD onset was defined as bacterial respiratory sample performed between 48h before ICU admission to ICU day-1.

SHR: subdistribution hazard ratio, $CI_{95\%}$ 95%: confidence interval, NIV: non-invasive ventilation, $PaCO_2$: CO_2 arterial partial pressure, PaO_2/FiO_2: O_2 arterial partial pressure/fraction of inspired O_2, SAPS2: simplified acute physiology score 2, SOFA: Sequential Organ Failure Assessment, CV: cardiovascular, eABT: early antibiotic therapy, COPD: Chronic obstructive pulmonary disease, AECOPD: acute exacerbation of COPD.
